# Numerical Study of Fin-and-Tube Heat Exchanger in Low-Pressure Environment: Air-Side Heat Transfer and Frictional Performance, Entropy Generation Analysis, and Model Development

**DOI:** 10.3390/e24070887

**Published:** 2022-06-28

**Authors:** Lei Zhang, Junwei Wang, Ran Liu, Guohua Li, Xiao Han, Zhiqiang Zhang, Jiayi Zhao, Baomin Dai

**Affiliations:** 1 Beijing Institute of Spacecraft Environment Engineering, Beijing 100094, China; zhangleicast@126.com (L.Z.); liuran_cast511@126.com (R.L.); li_guohua@foxmail.com (G.L.); hxcast@163.com (X.H.); 2 Tianjin Key Laboratory of Refrigeration Technology, Tianjin University of Commerce, Tianjin 300134, China; 21116030@bjtu.edu.cn (Z.Z.); zhaojy0204@163.com (J.Z.); dbm@tjcu.edu.cn (B.D.)

**Keywords:** negative air pressure, entropy generation, plain fin, heat exchanger, heat transfer coefficient, computational fluid dynamics (CFD)

## Abstract

Heat transfer and frictional performance at the air-side is predominant for the application and optimization of finned tube heat exchangers. For aerospace engineering, the heat exchanger operates under negative pressure, whereas the general prediction models of convective heat transfer coefficient and pressure penalty for this scenario are rarely reported. In the current study, a numerical model is developed to determine the air-side heat transfer and frictional performance. The influence of air pressure (absolute pressure) is discussed in detail, and the entropy generation considering the effect of heat transfer and pressure drop are analyzed. Furthermore, prediction models of air-side thermal and frictional factors are also developed. The results indicate that both the convective heat transfer coefficient and pressure penalty decrease significantly with decreasing air pressure, and the air-side heat transfer coefficient is decreased by 64.6~73.3% at an air pressure of 25 kPa compared with normal environment pressure. The entropy generation by temperature difference accounts for the highest proportion of the total entropy generation. The prediction correlations of Colburn *j*-factor and friction factor *f* show satisfactory accuracy with the absolute mean deviations of 7.48% and 9.42%, respectively. This study can provide a reference for the practical application of fined tube heat exchangers under a negative pressure environment.

## 1. Introduction

The finned tube heat exchanger is the main component of the cooling system and has been applied in versatile fields such as heating, air conditioning, and refrigeration for residential, industrial, commercial, and aerospace applications [1]. Most of the heat exchangers are designed for heating or cooling systems at normal atmospheric pressure. Meanwhile, the heat transfer coefficient on the air-side of the finned tube heat sink is relatively low compared to that of the tube-side, because of poor thermal properties of airflow. Therfore, the air-side thermal resistance strongly affect the heat exchange capacity of finned tube heat exchangers. For this reason, numerous attempts have been devoted to enhancing heat transfer at the air-side of finned tube heat exchangers [2], such as plain [3], wavy [4], and spiral [5] fin, helical wires [6], vortex generators and so on [7,8].

Han et al. [9] proposed three kinds of arc-winglet vortex generators and simulated the heat transfer and flow process across the fin. The front arrangements of VGs can be more effective in low *Re* zones. Based on the topology optimization method, Liu et al. [10] carried out a comparative investigation between optimized strip fins and traditional plain fins. The thermal performance of heat sink with optimized fins can be improved by approximately 22.64~28.04% when compared with the traditional structures. Che et al. [11] studied row-by-row heat transfer coefficient degradation by increasing tube rows with the staggered and in-line arrangement, the results from the comparative study indicate that the tube arrangement; airflow velocity, and row numbers have a significant impact on heat transfer coefficient. He et al. [12] numericaly investigated the finned tube heat exchanger with different vortex generator arrays; the results show that the heat exchange ability improved with the increasing attack angle of winglets; a significant augmentation of up to approximately 33.8~70.6% was yielded. Erek et al. [13] utilized 3D CFD modeling and parametric simulations to explore the influence of geometric parameters on thermal-hydraulic behavior of plate finned tube heat exchangers. It was found that fin tubes at the downstream region contribute to high heat exchange efficiency. Based on the local and global energy balances analysis method, Cobian-Iñiguez et al. [14] developed a 3D model to study the characteristic of compact finned tube heat sink with six tube rows. Their study emphasized the influence of operating conditions and the geometry and reporting changes in flow velocity considerably affected temperature fields and thermal performance.

In recent years, CFD has been widely employed in assessing heat transfer and flow phenomena relevant to heat exchanger design or evaluating the performance before the experimental testing [15], given that numerical modeling is reasonably verified. Välikangas et al. [16] conducted optimization of the plain fin-and-tube heat exchanger with fin pitches Fp = 1.5 mm and 3.5 mm utilized in the marine environment based on parametric CFD study, they concluded that new tube arrangement ratio Pt/Pl ≠ 1.1547 may offer higher efficiency. Yaïci et al. [17] conducted a numerical work to predict the effect of inlet airflow maldistribution on the thermal–hydraulic performance of plain finned tube heat exchangers. Considering inlet airflow velocity and flat-tube aspect ratio value, Alnakeeb et al. [18] numerically investigated the air-side performance; the results show that the pressure drop, respectively, decreased by 33.7% and 57.3% when decreasing the flat tube aspect ratio from 1 to 0.33 with 0.5 and 3.5 m/s inlet air velocity. Liu et al. [19] numerically analyzed heat enhancement of the heat exchanger with wave fin, considering fin pitch, wave-length and slits’ height; the research was finished through CFD simulation, through which the optimized structural parameters were obtained and a corresponding improvement of the heat transfer coefficient up to 34.2% was achieved. Using COMSOL Multiphysics software, Kalantari et al. [20] conducted a simulation to research the effects of geometric parameters on the conjugate heat transfer property of fin and tube heat exchangers; then, the corresponding heat transfer analytical correlations were developed. Lindqvist et al. [21] investigated the effect of the tube bundle array angle on the *j*/*f* ratio a semi-infinite finned tube heat sink, they reported that the results produced by Low Reynolds turbulence models are identical to ones from the laminar flow assumed model. Xie et al. [22] researched plate-and-fin air-side thermal and friction characteristics using CFD methods, empirical correlations for certain geometric parameters have been studied; it is revealed that the error of calculation results are within 20%. Tang et al. [23] researched the effects of different fin patterns. The corresponding correlations considering *Re* for five different fin patterns were fitted. Wang et al. [24,25] reported that the heat transfer is significantly impacted by the fin pitch for one and two tube-rows. With measured data, they fitted the empirical correlations of heat transfer and friction factor with average deviation of 7.5% and 8.3%, respectively. However, these correlations are based on limited case data and only valid for normal pressure environment, so it is ambiguous whether these theories can be applied for low-pressure conditions. Consequently, a new mathematical model should be developed to deeply study the heat transfer and frictional property of the air-side of finned tube heat exchanger for the working condition below normal air pressure.

Up to now, studies on heat exchangers operating at negative pressure were also conducted by some researchers. Jia et al. [26,27] experimentally and numerically studied the effects of low pressure environment on the air-side heat exchange and flow properties of a plate-fin heat exchanger. It is reported that, compared with atmospheric pressure, the thermal and flow performance is dramatically changed under low pressure environment. Li et al. [28] performed an experimental and numerical study on the corrugated fin radiator, and it is revealed that the convective heat transfer coefficient of the air-side decreased by 34% at −44 kPa in comparation to atmospheric pressure. Wan et al. [29] studied the air-side heat transfer and flow performance of louvered fin heat exchangers. These works indeed provide a valuable reference for heat transfer enhancement under negative pressure. However, the study in terms of impact of negative pressure on the performance of exchanger is not systematically conducted. Furthermore, general models to predict the convective heat transfer coefficient and frictional factor for the applications of negative pressure considering the influence of pressure and geometric are rarely reported.

Although the larger *j* factor or lower *f* factor means a better heat exchange performance or less pressure loss, these requirements cannot be met simultaneously due to their asynchronism. Thus, the entropy generation minimization proposed by Bejan [30,31] can be employed to evaluate the comprehensive performance of the heat exchanger as a guide to obtain the optimum results from research. Liu et al. [32] performed a numerical research on a fin-and-flat tube heat exchanger, the entropy generation is derived with numerical data for performance optimization. Zhou et al. [33] proposed a model of the plate heat exchanger with entropy generation minimization and obtained optimized geometric parameters. Considering the entropy production rate in heat exchangers, Sahiti et al. [34] performed an analysis on the variation of the entropy generation with *Re*. The results show that shorter flow lengths are accompanied by lower entropy production rates. However, little work was reported that was related to the entropy generation of a finned tube exchanger in negative pressure.

Overall, it can be concluded from the mentioned papers that the air-side heat transfer enhancement has been significantly improved by using different techniques. However, most of the research was carried out at normal atmospheric pressure, and the relevant prediction models under negative pressure are rarely developed and reported. In particular, for the applications of aerospace engineering, the pressure of the air is smaller than the normal atmosphere pressure. Thus, it is essential to research the air-side thermal and pressure drop properties and develop suitable models of fin-and-tube heat exchangers in negative pressure.

In this paper, to explore the thermal and friction performance at negative pressure, a numerical study of a plain fin-and-tube heat exchanger is performed, and the thermal-hydraulic performance of the heat exchanger under various low-pressure environment working conditions are systematically analyzed. In addition, entropy generation is applied to evaluate the heat exchange performance and pressure loss. Finally, the prediction models of *j* and *f* considering geometrical parameters and air pressure are developed. This study can provide theoretical guidance for the practical application of the finned tube heat exchanger used under the condition of negative pressure.

## 2. Model Description

### 2.1. Physical Model

**Figure 1:**
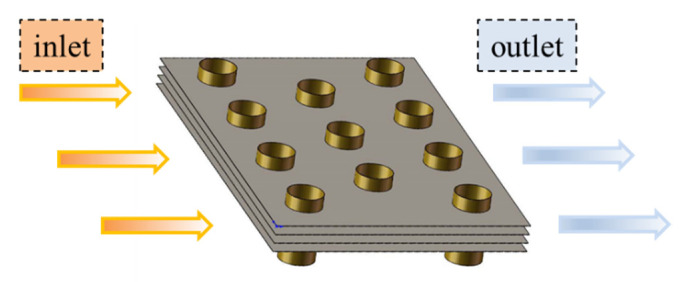
Schematic diagram core region of the compact finned tube exchanger.

Figure 1 depicts the schematic diagram for the core section of a plain finned tube exchanger, and the detailed geometric parameters of computational conditions are listed in Table 1. The tubes’ layout is in the form of a staggered arrangement, which can enhance the heat exchange process by changing the airflow direction [35,36]. The fin material is aluminum, of which the density and thermal conductivity are 2700 kg/m^3^ and 237.2 W/m·K, respectively. The air pressure ranges from 1 kPa to normal atmosphere pressure, and the inlet airflow velocity ranges from 0.5 to 6 m/s.

**Figure 2:**
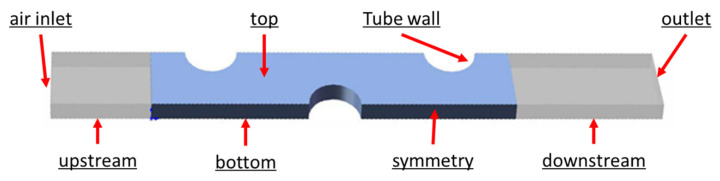
Computational domain for the simulated fin.

Figure 2 is an illustration of the simulated 3D computational domain. In order to ensure the uniform distribution of inlet fluid flow and avoid the outlet flow recirculation, the downstream and upstream of the computational domain were extended along the flow direction [37,38].

### 2.2. Governing Equations and Boundary Conditions

For the multi-physics system that couples flow and heat transfer: The fluid is considered to be incompressible due to different pressure conditions; the thermal contact resistance and heat radiation are ignored.

Based on these assumptions, the governing equations are shown as follows:

- Continuity equation:
  (1) $$\frac{\partial u_{i}}{\partial x_{i}}=0$$
- Momentum conservation equation:
  (2) $$\frac{\partial }{\partial x_{i}}(u_{i}u_{k})=\frac{\mu }{\rho }\frac{\partial }{\partial x_{i}}\left(\frac{\partial u_{k}}{\partial x_{i}}\right)-\frac{1}{\rho }\frac{\partial p}{\partial x_{k}}$$
- Energy conservation equation:
  (3) $$\frac{\partial }{\partial x_{i}}(u_{i}T)=\frac{k}{\rho c_{p}}\frac{\partial }{\partial x_{i}}\left(\frac{\partial T}{\partial x_{i}}\right)$$

**Figure 3:**
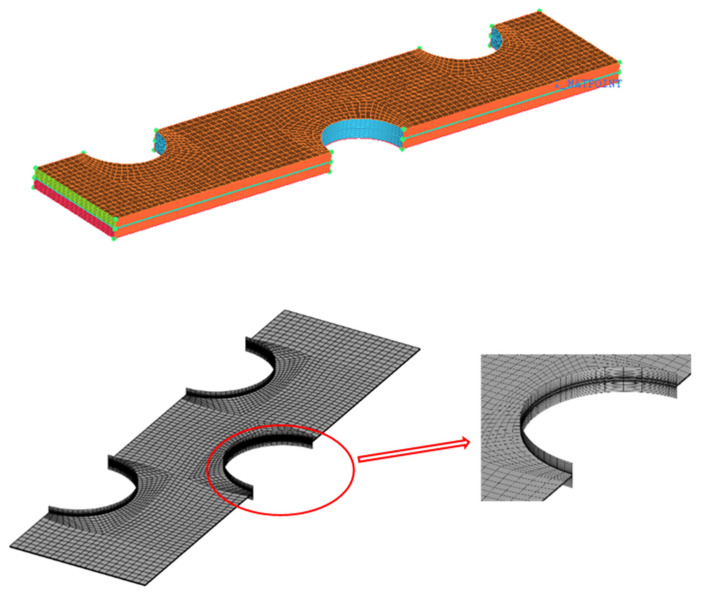
Computational grid generated in this work.

The the tube walls had a constant surface temperature of 203.15 K, the same as the evaporation temperature of the air conditioning system for aircraft. Symmetric boundaries were set as the left and right surfaces. The top and bottom surfaces were set as periodic boundaries. The inlet of the airflow was set as velocity inlet with a velocity of 0.5~6 m/s. The inlet temperature of the air was 213.15 K, in agreement with the temperature in space. The outlet of air was set as outflow. Structured meshes are utilized for the fin coil, described in Figure 3, ones nearby the fins and tubes surfaces were refined to reflect the coupled heat transfer flow performance between the air and walls.

### 2.3. Numerical Methods and Grid Independence Validation

**Figure 4:**
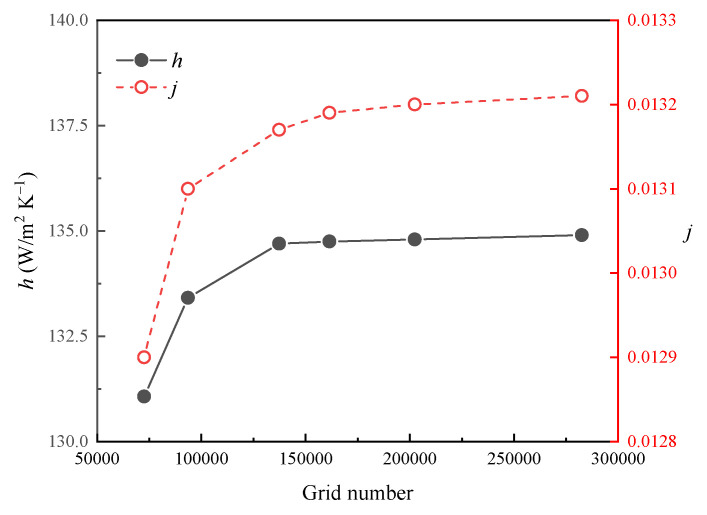
Mesh sensitivity analysis.

In this study, FLUENT 19.2 is employed to solve the governing equations based on the finite volume approach with structured meshing. The turbulent model standard k-ε of is employed in this simulation. A simple algorithm is applied to the iteration procedure. The second-order discretization scheme is employed for a high accuracy outcome. The numerical convergence criterion is accepted only when the residuals of velocities, pressure, temperature are smaller than 10^−6^ and 10^−4^ for continuity.The meshing model independence was verified, the grid number ranges from 72,566 to 282,594, and the calculation was conducted at the inlet air velocity of 3 m/s, and the data are shown in Figure 4. With the increasing quantity of grids, the Colburn *j*-factor and friction factor *f* rise rapidly at first and become stable. The number of structured grid increase from 161,538 to 282,594, and the difference in averaged calculation results is below 0.2%. Thus, the mesh with 161,538 cells was finally adopted to the simulations.

### 2.4. Parameter Definitions

The *Re* can be employed to describe the given flow conditions based on the double fin pitch by giving a measure of the ratio of inertial forces to viscous forces and consequently quantifies the relative importance of these two forces [39].

(4) $$Re=\frac{\rho u_{m}D_{c}}{\mu }$$

where *D_c_* is the collar tube diameter.

Meanwhile, the thermal and flow performance can be expressed by the *j* and *f*, respectively. The equations are defined as follows:

(5) $$j=\frac{h}{\rho u_{m}c_{p}}\times Pr^{2/3}$$

(6) $$f=\frac{\Delta p}{\frac{1}{2}\rho u_{m}^{2}}\times \frac{A_{c}}{A_{0}}$$

### 2.5. Model of Entropy Analysis

Assuming the thermal system consists of air (high -temperature heat source) and tube bundles (low-temperature heat source). According to thermodynamics theory, the entropy generation of air-side is expressed as:

(7) $$\Delta S_{1}=mc_{p}\ln\left(\frac{T_{2}}{T_{1}}\right)-mR\ln\left(\frac{p_{2}}{p_{1}}\right)=\frac{Q}{\Delta T}\ln\left(1+\frac{\Delta T}{T_{1}}\right)-mR\ln\left(1-\frac{\Delta p}{p_{1}}\right)$$

In above equations, *T*_1_ and *T*_2_ are the inlet and outlet temperature; *p*_1_ and *p*_2_ are the inlet and outlet pressure; *R* is gas constant. Equation (7) presents the entropy generation on the side of low-temperature heat source:

(8) $$\Delta S_{2}=\frac{Q}{T_{w}}$$

Thus, the total entropy generation can also be expressed as:

(9) $$\Delta S=\frac{Q}{\Delta T}\ln\left(1+\frac{\Delta T}{T_{1}}\right)-mR\ln\left(1-\frac{\Delta p}{p_{1}}\right)+\frac{Q}{T_{w}}\;=\;\Delta S_{T}+\Delta S_{P}$$

where the ∆*S_T_* and ∆*S_P_* are the entropy generation generated by heat transfer temperature difference and pressure penalty, respectively.

(10) $$\Delta S_{T}=Q\times \left[\frac{1}{\Delta T}\times \ln\left(1+\frac{\Delta T}{T_{1}}\right)+\frac{1}{T_{W}}\right]$$

(11) $$\Delta S_{P}=-m\times R\times \ln\left[1-\frac{\Delta p}{p_{1}}\right]$$

## 3. Model Validation

**Figure 5:**
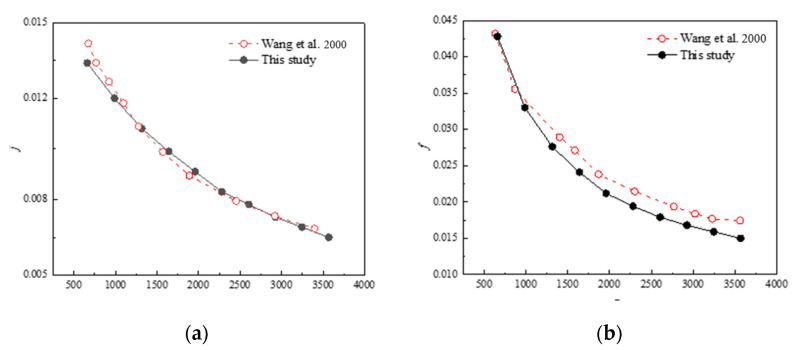
Comparison between calculated and experimental datas of Wang et al. [25]. (**a**) Colburn *j*-factor; (**b**) Friction factor *f*.

To ensure the reliability of the proposed numerical model and methodology, numerical simulation was carried out on a heat exchanger with identical geometric parameters as presented in the experimental study of Wang et al. [25], and the computational data were compared with the experimental results from mentioned literature. The inlet air velocity varies from 0.5 m/s to 2.5 m/s and the corresponding *Re* ranges from 600 to 3600. Figure 5 presents the *j* and *f* versus the *Re*. As one can see from the figure, the mean absolute error between the calculated Colburn *j*-factor and the experimental data is found to be 1.23%, and that of the friction factor is 8.42%. It is inferred that the error may be attributed to the airflow in the experiment being non-uniform while the uniform flow is assumed in the simulation. Nevertheless, almost all the error is relatively small and in the allowable range. The results of the numerical model are in good accordance with the experimental ones and this indicates the present model is dependable and can capture the heat transfer and flow friction mechanisms in fin-and-tube heat exchangers. Therefore, the model can be employed for further research.

## 4. Results and Discussions

### 4.1. Heat Transfer and Frictional Performance Analysis

To illustrate the results of the CFD calculation, based on selected environment pressure with the corresponding Reynolds numbers range from 55 to 5600 at an air velocity of 3 m/s, mainly velocity streamline, temperature and local pressure contours are plotted regarding the xy-plane of the upper surface of the computational domain.

**Figure 6:**
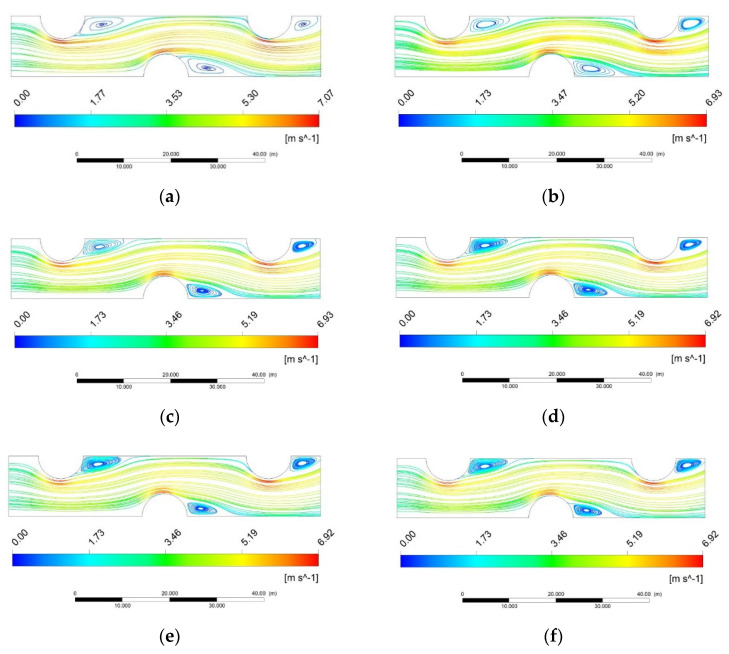
Velocity fields on the middle xy-plane with different pressure conditions. (**a**) 1 kPa (*Re* = 56); (**b**) 5 kPa (*Re* = 280); (**c**) 15 kPa (*Re* = 846); (**d**) 25 kPa (*Re* = 1413); (**e**) 45 kPa (*Re* = 2546); (**f**) 101 kPa (*Re* = 5600).

Figure 6 describes the velocity field of the air flows. It is found at the same inlet air velocity, higher environment pressure leads to larger *Re*. As known, according to the ideal gas state equation, the density is inversely proportional to the pressure at a constant temperature, the thinner air and slight viscosity in low pressure environment lead to lower *Re*. In Figure 6, flow zones named as wake zone with the lowest velocity are generated at the trailing edge. The maximum flow velocity is formed behind the tube walls facing the mainstream, which makes efficient heat exchange occur within this zone. Particularly, with the increase in environment pressure, due to the pressure difference between mainstream and wake zone, recirculation area horseshoe vortices are generated in the region behind the tube rows formed.

**Figure 7:**
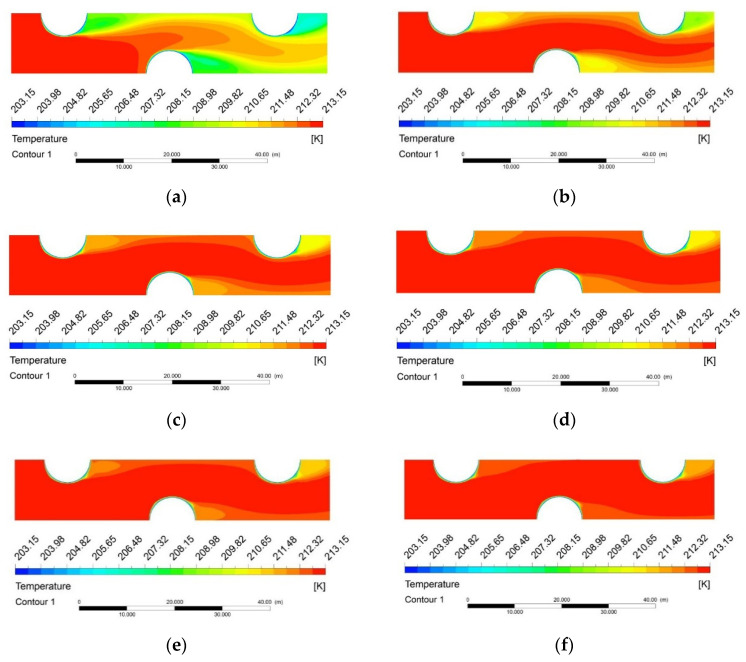
Temperature distribution on the middle xy-plane with different pressure conditions. (**a**) 1 kPa (*Re* = 56); (**b**) 5 kPa (*Re* = 280); (**c**) 15 kPa (*Re* = 846); (**d**) 25 kPa (*Re* = 1413); (**e**) 45 kPa (*Re* = 2546); (**f**) 101 kPa (*Re* = 5600).

The layered distribution of temperature field at environment pressures ranging from 1~101 kPa are shown in Figure 7. At the inlet airflow velocity of 3 m/s, the mainstream temperature at the same position in the flow field gradually increases with increasing pressure. The change is due to the enhancement of the thermal boundary layer under negative pressure. Temperature gradients are distributed in the boundary layer and the variation in the boundary layer at low pressure show a strong influence on the thermal and flow behavior [26]. Due to the impact of lower environment pressure on the physical properties of air, heat transfer is more intense when the air flows across the fin. As show in Figure 7a,b, when the environment pressure is higher, the temperature of the main flow region is much higher than that of the wake region. This is mainly attributed to that at higher Reynolds number, boundary layer separation occurs when it flows through the tube walls. It can be noted that, within wake region airflow the temperature is the lowest, and the temperature difference between the tubes and airflow is significantly low, which leads to a poor heat transfer performance in this region. It is also shown in Figure 7, the temperature gradient around tubes is higher at *p* = 101 kPa compared with that of *p* = 25 kPa, indicating a higher heat flux generated over the tubes and air, which leads to better heat transfer performance.

**Figure 8:**
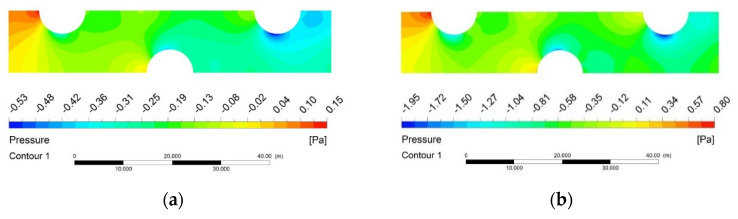

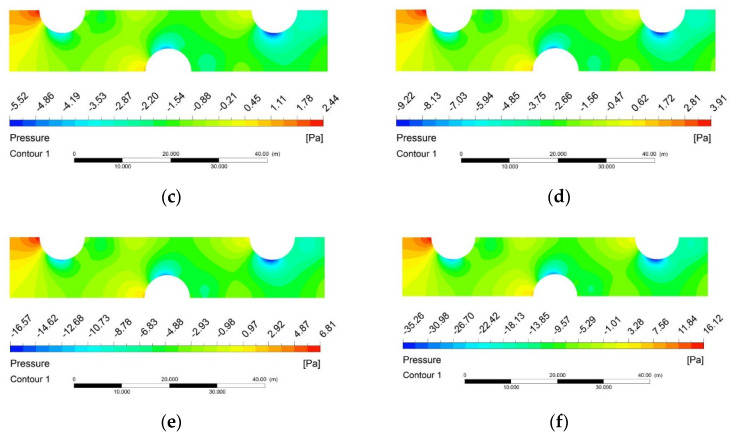
Air–side pressure field at different pressure conditions. (**a**) 1 kPa; (**b**) 5 kPa; (**c**) 15 kPa; (**d**) 25 kPa; (**e**) 45 kPa; (**f**) 101 kPa.

The air-side pressure field at different environment pressures are presented in Figure 8. As illustrated in the figure, the isobars are evenly distributed around the tube walls at low pressure. However, there are bigger pressure gradients around tubes when the air pressure is higher. It can also be seen that increasing environment pressure results in a larger pressure gradient along the longitudinal flow direction, and the local pressure drop as the airflow across the fin tubes is increased from 24.17 Pa to 98.33 Pa by changing the environment pressure from 1 kPa to 101 kPa. The main reason is that by keeping the same inlet air velocity, the increase of air density means an increase in *Re*, which leads to increasing of the convective heat transfer and friction performance.

**Figure 9:**
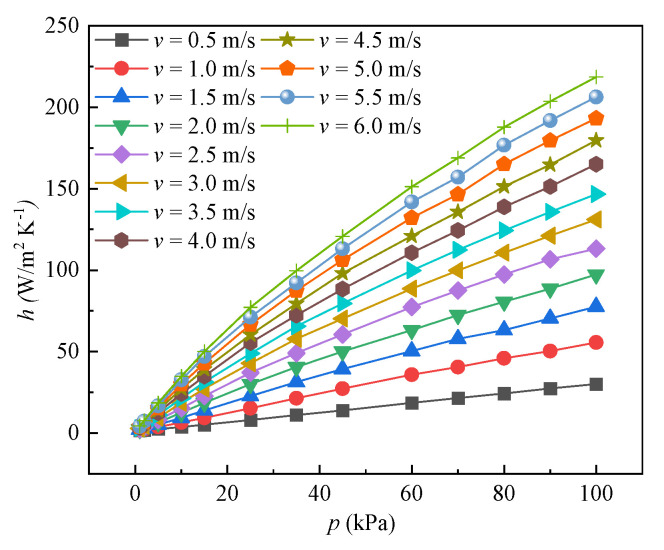
Variation of convective heat transfer coefficient under negative pressure conditions.

Figure 9 shows the effect of air pressure on the heat transfer coefficient *h* under different airflow velocities. As shown in the figure, the heat transfer coefficient decreases as the environment pressure decreases at the airflow velocity range from 0.5 m/s to 6 m/s. With increasing air pressure, *h* increases when the inlet air velocity is constant. According to the results of the simulations, h ranges from 8.02 to 77.26 W/m^2^ K at the air pressure of 25 kPa. In contrast, *h* ranges from 30.13 to 218.64 W/m^2^ K at atmospheric pressure of 101 kPa. Furthermore, it can be observed that the higher the airflow inlet velocity, the stronger the increase of *h*. For the case of inlet air velocity of 3 m/s, as the pressure decreases from 101 kPa to 0 kPa, the convective heat transfer coefficient on the air-side reduced by 32.4%, 67.25%, and 92.8% on average when the air pressure is 60 kPa, 25 kPa, and 5 kPa, respectively. It is indicated that the air-side convective heat transfer of fin-and-tube is dramatically deteriorated due to the thin air under low pressure. The density of air is positively related with the air pressure. Nevertheless, the specific heat capacity is not changed. Thus, the heat transfer capacity reduces when the airflow across the fin with the surrounding pressure decreases.

**Figure 10:**
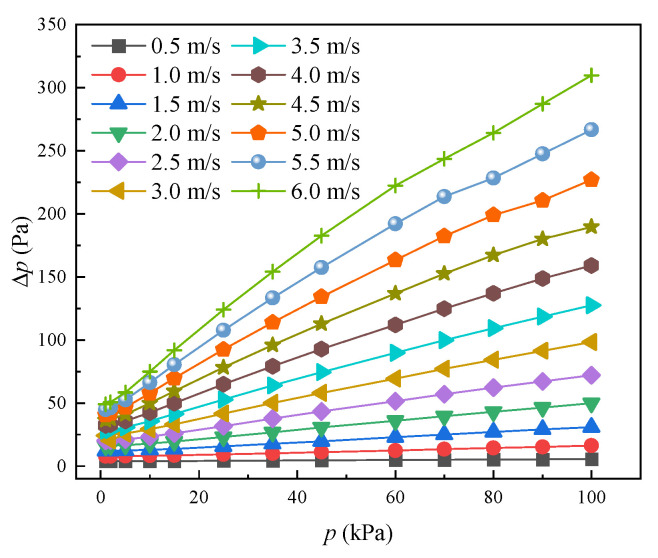
Pressure drop changing with air velocity at various air pressure.

Pressure drop is also an important indicator to assess the characteristics of a heat exchanger. The effect of air pressure on the pressure drop with various inlet airflow velocities is shown in Figure 10. The pressure penalty rises with the increase in the air pressure at the same velocity. When the environment pressure decreases from 101 to 1 kPa, the pressure drop decreases by 77.95% if the velocity is constant at 3.5 m/s. The reason is that the physical properties of airflow are sensitive to air pressure. When the air velocity increases from 3.0 to 6.0 m/s, the pressure drop increases by 202%, 220.5%, and 215.3% at an air pressure of 25 kPa, 60 kPa, and 101 kPa, respectively. It is demonstrated that the pressure drop increases more rapidly at higher velocities with air pressure increasing. It implies that mixed airflow due to larger *Re* at higher velocity contributes to the increase in pressure drop.

**Figure 11:**
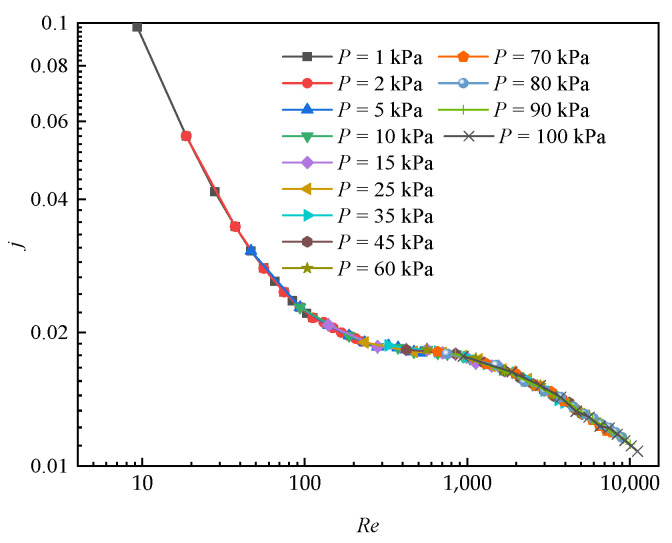
*j* versus *Re* at various negative pressure conditions.

Figure 11 illustrates the air-side *j* with *Re* for various environment pressure values. The Colburn factor was calculated by the Equation (5). As can be seen the higher the air pressure and *Re* numbers, the smaller the heat transfer factor. Despite different pressures, all the data nearly regresses to a certain profile as a function of *Re*. *j* under 25 kPa is increased by 28.15% in constrast to that of normal pressure. The main reason is that the heat transfer coefficient reduces at a slower rate than that of the density as the pressure decreases, so the *j* increases with decreasing pressure. In addition, it can be observed that *j* decreases dramatically at lower *Re*, because the thermalphysical properties of air vary more significantly when the environment is lower than 25 kPa. Thus, the heat transfer capacity is extremely small at a much lower pressure environment.

**Figure 12:**
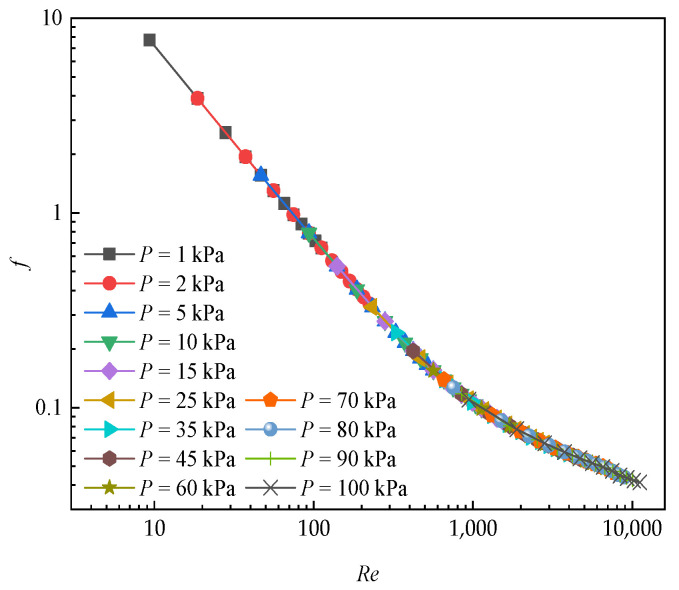
*f* versus *Re* at various negative pressure conditions.

Figure 12 depicates the variations in friction factor against Reynolds number under low pressure environment. It can be seen from the figure that the friction factor increases as *Re* decreases. In addition, it rises with gauge pressure reducing. *f* at 25 kPa is increased by 86.4% in comparison with the value at normal pressure, which indicates the pressure drop is lower at smaller air pressure. In addition, in the velocity range of 0.5~6 m/s, it is concluded that the friction factor, despite being under different air pressures, eventually fall on a certain curve that is closely correlated to *Re*. Additionally, the rapid decrease in *f* occurs when the when the environment is lower than 25 kPa, which is similar to the trend of *j* as shown in Figure 11.

### 4.2. Entropy Generation Analysis

The overall performance of the heat exchanger can be qualified using the entropy generation method, and the total entropy generation is due to heat transfer temperature difference and airflow pressure penalty, because entropy generation is proportional to degradation of available energy [2]. Accordingly, the effects of pressure conditions on entropy generation have been examined using the Entropy Generation—Pressure profile at the air pressure of 1~101 kPa.

**Figure 13:**
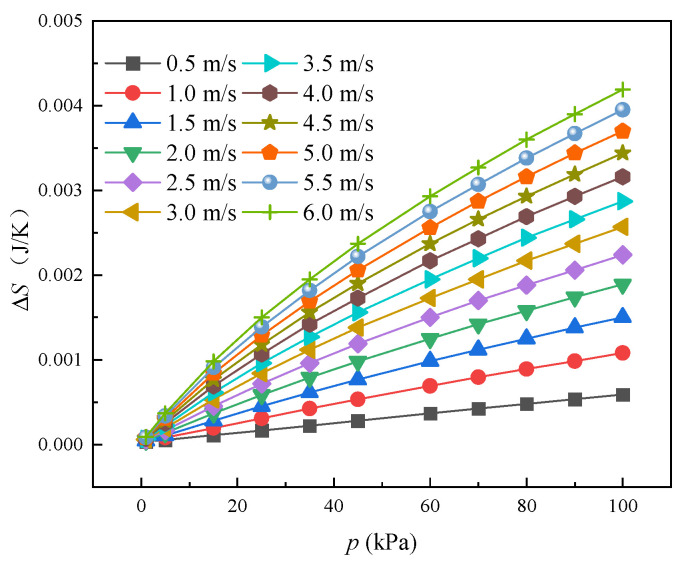
The effect of low pressure on the ∆*S*.

Figure 13 shows the variation in entropy generation with air pressure and airflow velocity. The entropy generation rises as the airflow velocity increases at the air pressure of 1 kPa to 101 kPa. Based on the simulated results, entropy generation increases about 205.8% as the air pressure rises from 25 to 101 kPa at airflow velocity of 3 m/s. At normal air pressure, the entropy generation can be increased by 610% when the wind velocity ranges from 0.5 m/s to 6 m/s. This is because that the average heat transfer temperature difference between air and tube wall increases with the enhancement of the air velocity, moreover, the pressure drop of the air-side also increases with the airflow rate. Furthermore, the air density also increases with the air pressure, so the specific entropy is also positive, relative to the air pressure. Thus, the entropy generation rises with the velocity and the air pressure.

**Figure 14:**
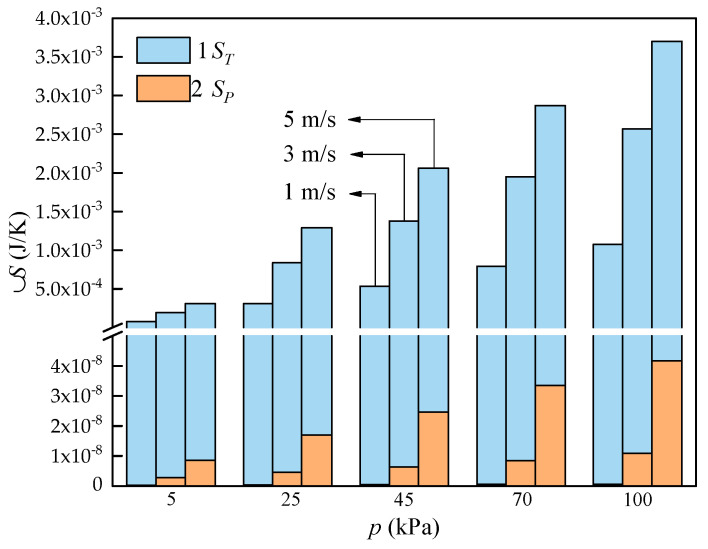
Statistical results of Δ*S_P_* and Δ*S_T_* at different air pressure.

The total entropy generation can be split into entropy generation by temperature difference Δ*S_T_* and entropy generation by pressure drop Δ*S_P_*. Δ*S_T_* and Δ*S_P_* at various environment pressure and air velocity are described in Figure 14. It can be found that both of them keep increasing with the uprising velocity and environment pressure of airflow. When the air pressure reduces from 101~5 kPa, Δ*S_T_* decreases by 92.52%, 92.44%, and 91.62% and reduction of Δ*S_P_* up to 50.58%, 73.86%, and 79.45% at the air velocities of 1, 3 and 5 m/s, separately. It is also attributed to the variation in thermal properties. In addition, keeping air pressure at 101 kPa, the entropy generation increased by 242.59% when the air speed ranges from 1 to 5 m/s, this due to the increment of airflow velocity leading to larger heat transfer temperature difference and flow resistance, resulting in the increase in heat exchange capacity and entropy generation. It can also be found that Δ*S_T_* is much higher than Δ*S_P_* under a certain working condition. For the cases discussed in this work, Δ*S_T_* accounts for above 99% of the total entropy generation while the entropy generated by pressure drop is really small. For instance, Δ*S_P_* accounting for 0.00011%, 0.00055% and 0.0013% at inlet air velocities of 1, 3 and 5 m/s, separately. Hence, it is concluded that during the design of a practical air-and-tube heat exchanger, the temperature difference between air and working fluid should be reduced to further reduce the entropy generation. Furthermore, considering the irreversible losses, air higher inlet air velocities are not recommended during the air-and-tube heat exchangers’ design and operation.

## 5. Model Development

Based on the data of simulated results, considering the effect of environment pressure and *Re* on the air-side heat transfer and pressure penalty, correlations to determine heat transfer and frictional performance are proposed. The models are developed using multivariate nonlinear regression techniques to quantify the effect of environment pressure.

Based on 144 cases with various operating parameters, the air-side heat transfer coefficient, *j* and *f* euqations with the *Re* and indicating the influence of environment pressure are proposed in this subsection. They are expressed as follows:

(12) $$h_{0}=0.2476Re^{0.7365}(p=101\;\mathrm{kpa})$$

(13) $$h=0.3967{\left(p/p_{0}\right)}^{0.0008}Re^{0.9107}Pr^{5.86}\left(0\;\mathrm{kpa}<p<25\;\mathrm{kpa}\right)$$

(14) $$h=2.2895{\left(p/p_{0}\right)}^{-0.0328}Re^{0.7816}Pr^{8.39}(25\;\mathrm{kpa}\;\leq p<101\;\mathrm{kpa})$$

where *p* is equal to the environment pressur, and *p*_0_ is the normal pressure of 101 kPa.

(15) $$j=0.0.4079Re^{-0.6127}{\left(p/p_{0}\right)}^{0.03231}\left(Re<200\right)$$

(16) $$j=0.04588Re^{-0.1425}{\left(p/p_{0}\right)}^{0.0134}\left(200<Re<11136\right)$$

(17) $$f=76.4078Re^{-0.99738}{\left(p/p_{0}\right)}^{-0.01076}\left(Re<500\right)$$

(18) $$f=2.8069Re^{-0.47}{\left(p/p_{0}\right)}^{-0.0063}\left(500\leq Re<11136\right)$$

**Figure 15:**
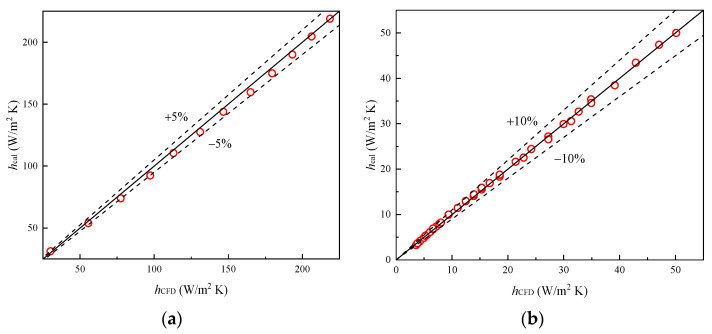

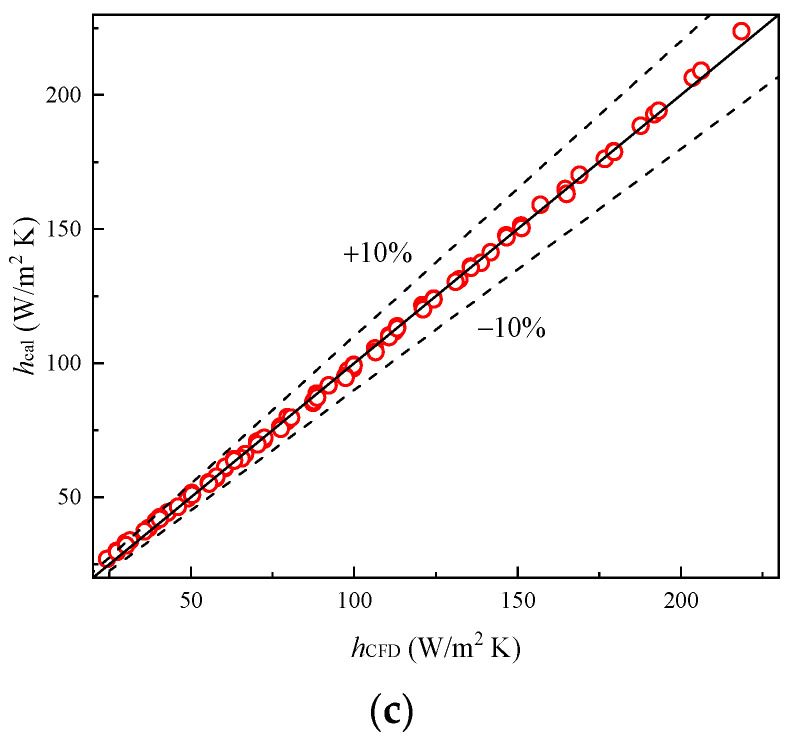
Correlation calculation error of *h.* (**a**) *h*_0_; (**b**) *h* (0 kPa < *p* < 25 kPa); (**c**) *h* (25 kPa ≤ *p* < 101 kPa).

Figure 15 illustrates the comparison of heat transfer coefficient between the simulation results and the newly proposed correlations. It can be noted that the newly developed model can predict all of the data within the ±5%, ±10%, and ±15% error range, respectively. Moreover, Equations (12)–(14) for the different pressure ranges give the mean deviation of 2.73%, 2.56%, and 1.66%, respectively.

**Figure 16:**
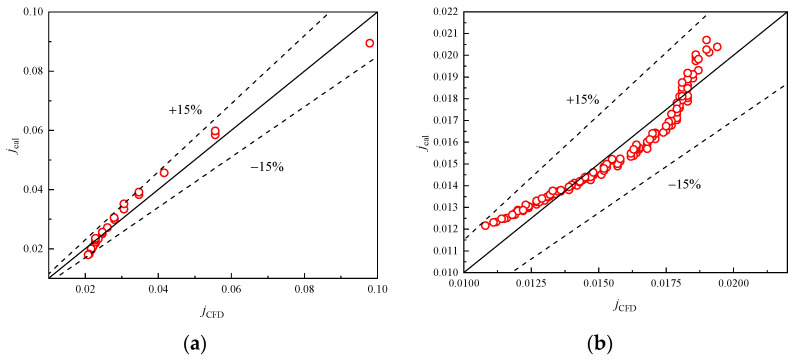
Correlation calculation error of Colburn *j*-factor. (**a**) *Re* < 200; (**b**) 200 < *Re* < 11,136.

**Figure 17:**
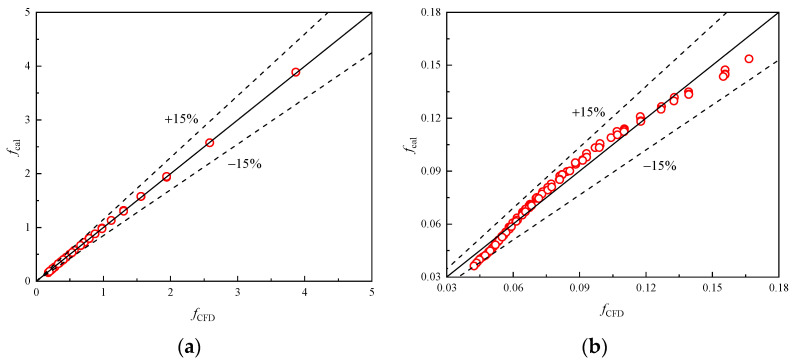
Correlation calculation error of friction factor *f*. (**a**) *Re* < 500; (**b**) (500 ≤ *Re* < 11,136).

The empirical correlations of *j* and *f* of the presently researched heat exchanger were fitted within the corresponding *Re* range. By using the correlation, the predicted and simulated data are shown in Figure 16 and Figure 17, respectively. The average deviations are 7.24% and 3.84% for *j*, and 2.78% and 5.42% for *f*, indicating that the newly proposed models are accurate and reliable. 

In order to widen the application range of the models, the heat transfer and friction factor correlations with *Re*, pressure conditions, and indicating the effect of geometric parameters including the various tube rows, fin pitch and tube collar listed in Table 2, more universal correlations, as expressed in Equations (19) and (20), are further proposed in this study. To develop such correlations, the simulations were undertaken considering the variation in geometric and working conditions. Based on the obtained 720 sets of data, the correlations were developed through a multiple linear regression method to determine the heat transfer coefficience and flow friction factor. The correlations are expressed as:

(19) $$j=0.2044\times Re^{-0.271}\times N^{-0.2903}\times {\left(\frac{F_{p}}{D_{c}}\right)}^{0.1143}\times {\left(\frac{p}{p_{0}}\right)}^{-0.029}$$

(20) $$f=17.6686\times \ln{\left(Re\right)}^{-3.0372}\times N^{0.2818}\times {\left(\frac{F_{p}}{D_{c}}\right)}^{-0.3053}\times {\left(\frac{p}{p_{0}}\right)}^{0.0198}$$

**Figure 18:**
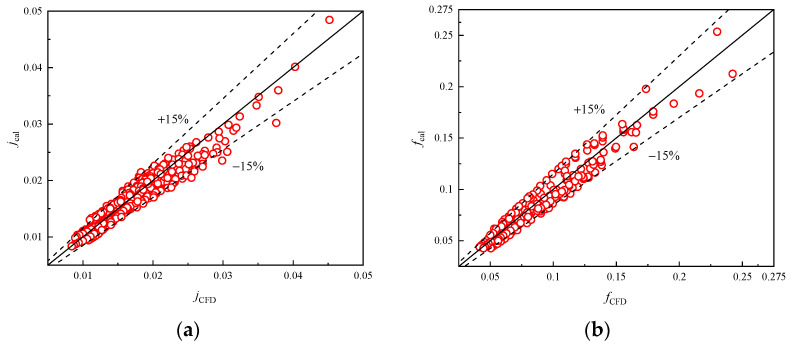
Verification of fitted correlations of *j* and *f* factor. (**a**) Colburn *j*-factor; (**b**) Friction factor.

Figure 18a,b illustrate the computational results and the correlation calculation results for the *j* and *f* factors, respectively. Good agreement between proposed correlations and numerical results is achieved in the range of 500 ≤ *Re_D_*_c_ ≤ 10,000. The error for 97.5% of results is within 15% and the absolute mean deviations of the correlation calculation results are 7.48% and 9.42%, respectively. This indicates that Equations (19) and (20) can describe precisely the heat transfer and flow mechanism in plain finned tube exchangers at low even extreme pressure. Meanwhile, when compered with relevant studies [28,29] in low pressure, the influences of tube rows and diameter are also considered in the present correlations. Thus, these correlations can be used for practical designs in practical engineering scenarios.

## 6. Conclusions

A CFD simulation of a plain fin-and-tube heat exchanger was developed and verified, aiming to evaluate the influence of the negative pressure on the air-side thermal and flow performance. Then, the overall performance of the air-side was evaluated based on the principle of entropy generation. Finally, air-side convective heat transfer coefficient and flow friction factor prediction models were developed. The conclusions are achieved as follows:

(1) The heat transfer and pressure drop behavior in the air-side of the exchanger has changed dramatically in the negative pressure environment. The temperature gradient around tubes decreases with the reduction in the air pressure. Moreover, the pressure gradient around tubes is larger at higher air pressure.
(2) The convective *h* and pressure drop reduced significantly when compared with ones at usual atmosphere pressure. At air pressure of 25 kPa, the *h* reduced by an average of 67.92% and pressure drop decreased by 53.45% on average when compared with that at 101 kPa.
(3) The entropy generation of the air-side increases with the increase in air pressure and airflow velocity. The entropy generation increases about 205.8% by increasing the air pressure from 25 kPa to 101 kPa.
(4) The entropy generation by temperature difference Δ*S_T_* accounts for the vast majority proportion of the overall entropy generation compared with that by pressure drop Δ*S_P_*. The temperature difference between the air and the refrigerant should be reduced to further reduce the entropy generation.
(5) The models of *j* and *f* at the plain fin air-side in environment with negative pressure are developed with a mean absolute error of 7.48% and 9.42%, respectively, which shows high accuracy.

## Figures and Tables

**Table 1:** Global parameters of the heat exchanger and computational conditions.

Parameter	Size or Value
Tube diameter (*D_c_*)	9.52 mm
Transverse tube spacing (*P_t_*)	25.4 mm
Longitudinal tube spacing (*P_l_*)	22 mm
Fin pitch (*F_p_*)	1.23 mm
Fin thickness (*δ_f_*)	0.1 mm
Frontal velocity (*v_in_*)	0.5~6 m/s
Wall temperature (*T_w_*)	203.15 K
Tube bank number (*N*)	3
Thermal conductivity of the fin (*λ*)	236 W m^−1^ K^−1^
Inlet temperature of air (*T_in_*)	213.15 K
Air pressure (*p*)	1~101 kPa

**Table 2:** Geometric parameters for simulation cases.

	*P_t_* (mm)	*P_l_* (mm)	*σ* (mm)	*D*_c_ (mm)	*N*	*F_p_* (mm)	*P_t_* (mm)
1	25	22	0.1	9.52	3	1.23	1
2				9.52	3	2.23	2
3				9.52	3	2.5	3
4				9.52	3	2.8	4
5				9.52	3	3.23	5
6				9.52	3	3.6	6
7				5	3	2.5	7
8				7	3	2.5	8
9				7.94	3	2.5	9
10				10.23	3	2.5	10
11				9.52	4	2.5	11
12				9.52	6	2.5	12

## Nomenclature

*A_c_*	minimum flow cross-sectional area (m^2^)
*A_f_*	fin surface area (m^2^)
*A_fr_*	frontal area (m^2^)
*A*_0_	total surface area (m^2^)
*D_c_*	tube outer diameter (m)
*D_h_*	hydraulic diameter, 4A_c_ * L/A_0_ (m)
*F_p_*	fin pitch (m)
*f*	friction factor
*h*	heat transfer coefficient (W m^−2^ K^−1^) *h* = *Q*/(A_f_*Δ*T*), or the height of the delta winglet (m)
*j*	Colburn factor
*L*	fin length along the main flow direction (m)
*l*	chord length of the large winglet (m)
*m*	massflow rate (kg/s)
*Nu*	Nusselt number
*p*	pressure (kPa)
Δ*p*	pressure drop (kPa)
*P_l_*	longitudinal tube pitch (m)
*P_t_*	transverse tube pitch (m)
*P_r_*	Prandtl number
*Re*	Reynolds number based on tube collar diameter
Q	heat transfer rate (W)
*p*	Pressure (kPa)
Δ*S*	entropy generation (J K^−1^)
Δ*S_T_*	entropy generation by temperature difference
Δ*S_P_*	entropy generation by pressure drop
*T*	temperature (K)
*T*_w_	wall temperature (K)
Δ*T*	Temperature difference (K)
*u*, *v*, *w*	velocity components in x-, y-, z- directions (m s^−1^)
*u*_m_	mean velocity at the minimum flow cross-sectional area (m s^−1^)
U	velocity vector (m s^−1^)
x, y, z	Cartesian coordinates (m)
Z	heat transfer power per unit temperature and per unit volume (W m^−3^ K^−1^)
**Greek Symbols**	
*δ*_f_	fin thickness (m)
*μ*	dynamic viscosity (kg m^−1^ s^−1^)
*ρ*	density (kg m^−3^)
*λ*	thermal conductivity (W m^−1^ K^−1^)
